# A Survey on Dietary Supplement Consumption in Amateur and Professional Rugby Players

**DOI:** 10.3390/foods10010007

**Published:** 2020-12-22

**Authors:** Antonio Jesús Sánchez-Oliver, Raúl Domínguez, Paola López-Tapia, Francisco Miguel Tobal, Pablo Jodra, Juan José Montoya, Eduardo J. Guerra-Hernández, Juan José Ramos-Álvarez

**Affiliations:** 1 Departamento de Motricidad Humana y Rendimiento Deportivo, Universidad de Sevilla, 41013 Sevilla, Spain; sanchezoliver@us.es; 2 Escuela Universitaria de Osuna (Centro adscrito a la Universidad de Sevilla), 41640 Osuna, Sevilla, Spain; 3 Departamento de Educación Física y Deporte, Universidad de Sevilla, 41013 Sevilla, Spain; 4 Studies Research Group in Neuromuscular Responses (GEPREN), University of Lavras, 37200-000 Lavras, Brazil; 5 Sport of Medicine Department, Universidad de la República de Uruguay, 11600 Montevideo, Uruguay; paola.lopezt@hotmail.com; 6 Faculty of Medicine, School of Medicine of Physical Education and Sport, Complutense University, 28040 Madrid, Spain; miguelto@ucm.es (F.M.T.); jjmontoya@ucm.es (J.J.M.); jjramosa@ucm.es (J.J.R.-Á.); 7 Faculty of Education Sciences, University of Alcalá, 19001 Guadalajara, Spain; pablo.jodraj@uah.es; 8 Department of Nutrition and Food Science, University of Granada, 18071 Granada, Spain; ejguerra@ugr.es

**Keywords:** supplementation, ergogenic aid, nutrition, football, sport team

## Abstract

Purpose: the aim of the present study was to analyse the pattern of dietary supplements (DS) consumption on federated rugby players, including the analysis of differences based on the sex and competitive level (professional vs. amateurs). Material and methods: 144 rugby players (83 male and 61 female), of whom 69 were professionals and 75 amateurs, were recruited for the study. All the participants filled out a specific questionnaire about DS consumption including questions related to the consumption of DS and their effects on sport performance and health status. Results: 65.3% of participants declared consuming at least one DS, with a higher prevalence in males than females (77.1% vs. 49.2%) and in professionals thanin amateur players (79.7% vs. 52.0%). The main reason for consumption was to enhance sport performance (62.3%) with differences only based on sex (74.3% males vs. 43.2% females). The most common purchase sites were the Internet (45.6%) and specialised stores (39.8%). As to the moment of ingestion, professionals did this most frequently during competition and training (56.4% vs. 28.2%), whereas amateur players did so only during competition (20.5% vs. 3.6%). Moreover, professional player intake most frequently in post-exercise (65.5% vs. 35.9%), whereas amateur during pre-exercise (30.8% vs. 5.5%). The DS most consumed included whey protein (44%), caffeine (42%), sports drinks (38%), energy bars (34%) and creatine monohydrate (31%), with a higher prevalence in male and professional players of whey protein and creatine monohydrate. Conclusions: The main reason for DS consumption is for enhancing sports performance). Professional players more frequently purchase them on the Internet and consume DS during training and competition period and in the post-exercise, whereas amateur players consume during competition and pre-exercise. Related to the main form of DS consumption, it is observed that a moderate consumption of DS could be considered ergogenic, such as whey protein, sport bar and creatine, while an absence of other DS could be considered ergogenic.

## 1. Introduction

Rugby has been gaining international popularity with approximately 8.5 million registered players in over 121 countries around the world [1]. This sport is played by two teams that compete during two thirty- or forty-minute halves with a ten-minute halftime, and with rest periods between matches of 4 to 7 days according to level [2,3,4]. As in other team sports, rugby performance is determined by the complex interplay of the physical, technical, tactical and cognitive qualities of each player [5]. Matches are intermittent and involve regular periods of high-intensity activity (e.g., high-speed running) interspersed with low-intensity activity, such as recovery from position or light running [6,7]. The total distance covered during the match ranges from 3500 to 8000 m, depending on the playing position and level of competition [8], including up to 1000 m ran at high speed [7] in efforts of approximately 10 m [9]. In addition to high-speed runs, players are frequently involved in many collisions and struggles due to high-intensity defensive (e.g., tackling) and offensive (e.g., ball carrying) actions [10].

Due to the great physiological demands of this sport, rugby players require a high development of anthropometric and physical qualities (linear speed, change of direction, endurance capacity, muscle strength and power) to perform at high performance [6,8,11]. In addition, it must be considered that the physiological aspects differ by sex, as the speed, agility, muscle power or maximum aerobic power estimated in women are lower compared to men [12], and that the demands of the game vary according to position and level of competition. The wings are involved in more free runs, pivots assume a greater decision making and ball handling functions and backs are involved in more physical collisions [8,11,13]. Therefore, the rugby player must have different qualities (strength, speed, power) to improve their performance in this sport [14,15]. This high demand [8,16], as well as the possibility that any small gain gained may provide a real improvement in the result in performance and competition [17], encourages athletes to consider the use of tools and/or strategies among which can be found the use of dietary supplements (DS) [18]. The International Olympic Committee (2018) defines a DS as a food, food component or nutrient that is intentionally ingested, in addition to the diet commonly consumed, with the aim of achieving a specific physical performance or a health benefit [19].

Despite the multitude of DS that we find on the market, only a few are supported by scientific evidence [20]. Looking to clarify this situation, several important international institutions have made recommendations through consensus or positions in which they collect the levels of evidence regarding the efficacy and/or safety of the different DS. Currently, three documents can be highlighted: (i) the document published every 4 years by the Australian Institute of Sport (AIS) [21]; (ii) the consensus of 2018 by the group of experts of the International Olympic Committee (IOC) [19]; and (iii) the document published by the International Society of Sports Nutrition (ISSN) from time to time [22]. These documents are constantly updated based on the increase in the number of scientific investigations that analyse the effect of the different DS in sports. Therefore, we are faced with dynamic documents that are being adapted and that help decision-making in the recommendations on the use of DS in sports, based on criteria of their safety, efficacy and legality [20].

The range of DS consumption in sports is very wide, and currently we place it between 30–95% [23]. This variation in consumption will depend on several variables, among which (i) the type of physical activity or sport, (ii) the level of performance or competition of the athlete, (iii) sex and (iv) age stand out [18,24,25,26,27,28]. Although studies on the prevalence and pattern of DS consumption can be found in rugby players of different levels of competition, age and/or sex [29,30,31,32,33] or included with other athletes in heterogeneous samples [34,35], to date, there is no research carried out exclusively on these athletes that studies their prevalence and consumption patterns based on the variables that determine it. Thus, the objective of the present study was to analyse the DS consumption pattern in federated rugby players, including possible differences based on sex and competitive level (professional vs. amateur).

## 2. Materials and Methods

### 2.1. Participants

A total of 144 rugby players (83 men and 61 women), of whom 69 were professionals (División de Honor A y Liga Iberdrola) and 75 amateurs (División de Honor B), voluntarily participated in the present study (see the characteristics of the sample on Table 1). All the participants belong to the Autonomic Community of Madrid’s delegation of the Spanish Rugby Federation. All of them went individually to the Professional Specialisation of Physical Education and Sports Medicine School (EEPMEFD being its acronym in Spanish) of the Complutense University of Madrid at the beginning of the 2019–2020 season for a medical examination. Once there, the objective and characteristics of the study were explained, and the consent of all the participants was obtained. This research project has the approval of the ethics committee of the Alfonso X El Sabio University.

### 2.2. Experimental Design

For data collection, the athletes completed a questionnaire on DS consumption. The administration of the questionnaire took place individually. A person in charge of the EEPMEFD, an expert in the matter, was present during the completion to answer questions and direct the procedure and correct delivery of the responses.

### 2.3. Instrument

The questionnaire used was previously validated, based on its content, its application, its structure and its presentation [36]. In a review conducted by Knapik et al. [24] that assessed the quality of questionnaires aiming to determine the prevalence in the use of dietary supplements by athletes, this same questionnaire was rated, and it achieved 54% methodological quality. The methodological quality in the Knapik’s evaluation was rated by using an 8-point scale that included assessments for sampling methods, sampling frame, sample size, measurement tools, bias, response rate, statistical presentation and description of the participant sample. The percentage of methodological quality was obtained by the ratings obtained in all these characteristics of the questionnaire, and the current questionnaire was one of the 57 questionnaires (out of 164) reviewed that were considered suitable to obtain accurate information of supplement use by athletes. It is worth highlighting its use in different studies that have analysed DS consumption in athletes [18,23,26]. The questionnaire contains three main sections: the first collects the anthropometric, personal, and social data of the respondent; the second encompasses the practice of sports activity and its context; and the last and most extensive is related to DS consumption. This part includes, among other questions: what DS they consume, why they consume them (i.e., sport performance, health, esthetic), who advises them (i.e., doctor, dietitian-nutritionist, friends, family, physical trainer), where they buy them (i.e., pharmacy, Internet, specialized stores) and when they take them (before, during and after training, and/or competition) and the moment of consumption (training, competition, or both).

### 2.4. Statistical Analysis

The Kolmogorov–Smirnoff test and the Levene test were applied for checking the normality and the homoscedasticity. Quantitative variables are presented as an average (M) ± standard deviation (SD), while qualitative variables are in percentages. For the analysis of possible differences based on the level of performance (professional vs. amateurs) or sex about specific questions regarding the motivation, expectations and contextualisation of the use of DS, such as the prevalence of consuming DS with a level of at least 10% of the sample, a chi-square test (χ2) was performed. Additionally, if statistical differences were reported, an odds ratio (OR) was performed. As to the total DS ingested, a Student’s T-test for independent samples was carried out to analyse possible differences between the levels of performance or sex. The statistical level of significance was set at *p* < 0.05. The statistical analyses were performed using the Statistical Package for Social Sciences (version 18.0 for Mac, SPSSTM Inc, Chicago, IL, USA).

## 3. Results

Of the sample, 65.3% declared having ingested at least one DS on some occasion, and there were statistical differences by sex (*p* = 0.001) and performance level (*p* = 0.001). Thus, the proportion of males that consume DS was higher than females (77.1% vs. 49.2%; OR = 1.79 [1.22–2.63]), whereas a higher prevalence of ingesting DS in professional players than in amateur players was detected (79.7% vs. 52.0%; OR = 2.09 [1.29–3.38]). Regarding the sample’s average of DS consumption, this was 3.90 ± 3.56 supplements with a non-statistical higher consumption in males compared to females (4.44 ± 3.51 vs. 3.44 ± 3.57; *p* = 0.10) and professionals compared to amateurs (4.35 ± 3.84 vs. 3.30 ± 3.07; *p* = 0.08).

**Figure 1:**
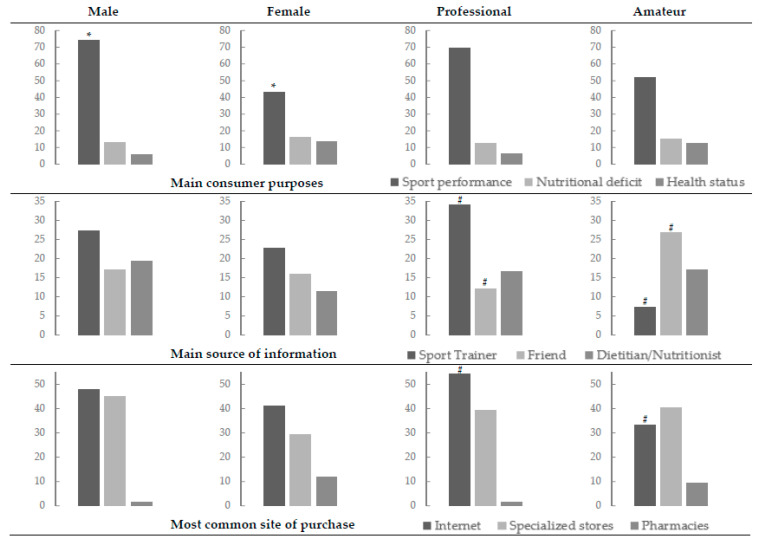
Main consumer purpose, source of information and site of purchase (sex and level of performance) (expressed as a percentage). * Relative risk of dietary supplement DS consumption of male vs. female players; # Relative risk of DS consumption of professional vs. amateur players.

The main purpose of DS consumption was to improve sport performance (62.3%), followed by the prevention of nutritional deficits (14.0%), to enhance health status (8.8%) and physical appearance (7.0%), with statistical differences based on sex (*p* = 0.002), but not on performance level (*p* = 0.205). Therefore, the proportion of males compared to females who consumed DS for enhancing sport performance was higher (74.3% vs. 43.2%; OR = 1.75 [1.19–2.57]) (see Figure 1).

As to the main source of information to determine the type, use, and utility of DS, 25.8% declared that this was advised by a sport trainer, 16.7% by friends or dietitians/nutritionists, 13.6% by coaches and 12.1% by teammates, with statistical differences between the sex (*p* = 0.010) and the level of the players (*p* = 0.009). Thus, on the one hand, the males were advised in higher proportions than females by a sport trainer (27.3% vs. 22.7%), dietitians/nutritionists (19.3% vs. 11.4%), friends (17.0% vs. 15.9%) and teammates (13.6% vs. 9.1%), whereas the females were more advised than the males by doctors (15.9% vs. 0%) or coaches (15.9% vs. 12.5%); on the other hand, professional players were more advised by a sport trainer (34.1% vs. 7.3%; OR = 1.49 [1.23–1.80]), while amateur players were advised more frequently by friends (26.8% vs. 12.1%; OR = 1.83 [1.09–3.09]) and doctors (12.2% vs. 2.2%; OR = 2.48 [1.43–4.29]) (see Figure 1).

The most common purchase site of DS was the Internet (45.6%), followed by specialised stores (39.8%), pharmacies (4.9%) or other places (9.7%), without differences between sex (*p* = 0.105) and a trend to statistical differences based on the level of the players (*p* = 0.059). Therefore, the professional players obtained DS more frequently on the Internet (54.1% vs. 33.3%; OR = 1.40 [1.01–1.94]), while purchases in specialised stores were similar (39.3% vs. 40.5%) (see Figure 1).

Regarding the moment of consumption, 44.7% of the sample declared ingesting DS during training and competition, while 29.8% affirmed ingesting them only during training, 10.6% during the competition days, 8.5% every day of the year and 6.4% occasionally. As to the moment of consumption, no effect was reported for sex (*p* = 0.22), but differences based on competition level were found (*p* = 0.020), with a higher proportion of professional compared to amateur players taking DS during training and competition (56.4% vs. 28.2%; OR = 1.60 [1.13–2.27]), whereas amateur players consume DS more frequently only during competition (20.5% vs. 3.6%; OR = 2.17 [1.42–3.31]).

Concerning the timing of the supplementation intake, that most frequent was post-exercise (53.2%), followed by pre-, during- and post-exercise (17.0%), pre-exercise (16.0%) and indifferently (5.3%). Statistical differences were observed based on the level of competition (*p* = 0.004), but not sex (*p* = 0.184). Thus, a higher proportion of professional players who ingest DS post-exercise (65.5% vs. 35.9%; OR = 1.67 [1.13–2.45]) and pre-, during- and post-exercise (20.0% vs. 12.8%) was detected, while amateur players more frequently ingested DS pre-exercise than professional players (30.8% vs. 5.5%; OR = 2.34 [1.57–3.50]).

The most consumed DS was whey protein (44%) followed by caffeine (42%), sport drinks (38%), energy bars (34%), creatine monohydrate (31%), Branched-Chain Amino Acids (BCAAs) (19%) and glutamine (12%) (see Table 2). Regarding sex, a higher prevalence of consumption was reported in male players of whey protein (*p* < 0.001; OR = 1.76 [1.32–2.35]), creatine monohydrate (*p* < 0.001; OR = 1.69 [1.30–2.18]) and glutamine (*p* = 0.036; OR = 1.52 [1.15–1.99]); whereas, concerning the level of performance there was a higher consumption in professional players compared to amateur players of whey protein (*p* < 0.001; OR = 2.57 [1.76–3.76]), creatine monohydrate (*p* = 0.011; OR = 1.60 [1.15–2.21]) and BCAAs (*p* < 0.001; OR = 1.90 [1.41–2.56]).

## 4. Discussion

The objective of the present study was to analyse the DS consumption pattern in rugby players, including possible differences based on sex and the competitive level (professional vs. amateur). Although there are different studies that analyse the consumption of supplements and other substances in rugby players [30,31,32,33,34,35], this study is the first that analyses the consumption of DS in Spanish rugby federated athletes according to sex and the level of competition.

Of the total sample, 65.3% reported the use of at least one DS. These data are similar to those reported in Spanish athletes of different sports modalities (64%) [27] and Irish school rugby players (65%) [29], but higher than those obtained in rugby players from Uganda (13.4%) [34] and Kenya (55.1%) [31] and lower than the data reported in a study that was carried out with 166 elite players of the European Super League (95%) [37]. These differences may be due to the strong influence of perceived cultural norms (both sporting and non-sporting), and the popularity and status of rugby in the different countries studied [19,29]. It should be noted that the results found in the present study support the hypothesis that suggests both an increased DS consumption with a higher level of training or competition and a higher consumption in males compared to females [19], two of the variables that most influence the consumption of DS [24,38]. Furthermore, these results are similar to those reported in different studies that have detected a higher consumption in males [18,27,38] and athletes with a higher level of competition [18,24,26,39,40].

The main reason for DS consumption in the sample was to enhance sport performance (73.2%), a result similar to that which was found in Spanish athletes of other modalities (45–77.8%) [18,26,27,39]. These data also match those reported by Woolfenden (2017), in which it was found that performance improvement was the main motivation for DS consumption in rugby players, reaching 90% [37]. The lower rate reported in our results (73.2%) compared to Woolfenden (2017) could be the level of the players, since the latter analysed a sample of 166 elite players from the European Super League, one of the most competitive leagues in the world. The results found in this study support that there may be differences in the purpose of DS consumption by sex, the most common purpose in men being the improvement of performance or physical appearance and in women health or prevention of nutritional deficits [18,26,39,41].

The source of information that determines the use of DS in sport is very important because it has been proven that athletes’ knowledge about DS is low [29,39,42,43]. Although the advice for DS consumption should come from experts in the field (dietitians/nutritionists or sports medicine doctors) [44], the data obtained do not support this, since only 13.6% of the total sample was advised by a dietitian or nutritionist and only 15.9% of the women were advised by a doctor. In addition, although there were also differences in the main source of information depending on the level of competition, physical trainers were those most chosen by amateurs, and friends by professional players. These data match the results found in amateur rugby players, in which the main sources of information were not experts in the matter [29] but are the opposite to those reported in elite rugby players of the European Super League who were advised by dietitians or nutritionists (73%) and sports scientists (39%) [37]. The data obtained show the need to influence the correct sources of advice for the DS, since bad information and bad advice can lead to the consumption of a DS without evidence or, what is worse, to a case of unintentional doping and a health risk [45,46]. This is aggravated in this sport, which is among those with the highest incidence of doping, defining DS as a possible gateway to doping [30,32]. In addition, it should be noticed that athletes who receive advice from a dietitian/nutritionist as the main source of nutritional information have better eating habits, a higher understanding of the periodization of nutrients [47] and a consumption of DS with a high level of scientific evidence on its performance-enhancing effect [44].

The stores where athletes purchase the DS is another issue that can cause malpractice [23,48]. In relation to this, the data obtained in the present study support this theory, given that the DS purchasing preferences for the sample were mostly online (45.6%), which was more frequent in professional players (54.1% vs. 33.3%; OR = 1.40 [1.01–1.94]). Purchasing DS in this way may lead to health and/or performance risks due to biased and unreliable information from this source [27,29,34], discovering pharmacological substances not declared on the label [49,50], which is inappropriate with respect to the optimal protocol [51,52,53], or lack of specific legislation [45,50]. To enhance control and monitorisation, it is necessary to promote a specific legislation about DS in sport, as many studies have revealed that consumers are unaware of what they are taking and are even consuming DS or ingredients included in them that are prohibited or harmful to health [45,54,55].

The period of consumption of DS is another aspect to consider in their use. Thus, in addition to the amount, the moment of ingesting may influence the possible ergogenic effect [56]. Regarding this, the highest percentage of the sample declared consuming DS during training and competition (44.7%). An increase in the level of competition implies a greater demand in all aspects, both in training and in competition [17]. This, as well as the high demand of this sport [8,16] and the pressure in professional rugby [32,37], may justify the difference between the period of consumption of professionals, who use them more frequently in training and competition (56.4% vs. 28.2%; OR = 1.60 [1.13–2.27]), compared to amateurs, who tend to consume them more frequently only during competition (20.5% vs. 3, 6%; OR = 2.17 [1.42–3.31]. Regarding the timing or moment of ingesting depending on exercise, statistical differences were also found depending on the level of competence (*p* = 0.004), but not on sex. Thus, professional players ingested DS more frequently post-exercise (65.5% vs. 35.9%; OR = 1.67 [1.13–2.45]), compared to amateurs, who did so more frequently pre-exercise (30.8% vs. 5.5%; OR = 2.34 [1.57–3.50]).

The DS most consumed by the present sample were whey protein, caffeine, sport drinks, sport bars and creatine monohydrate. Observing these results, we can find differences between the DS consumed by sex and performance level, with significant differences by sex in protein, creatine monohydrate and glutamine, and by level of performance in protein, creatine monohydrate and BCAAs. Consistent with this study, there are other studies in which male [37,57,58,59] and higher-performance athletes [26,37] opt for protein supplements such as whey protein, amino acids or creatine, among other DS, because the main reasons for supplementation were related to improving overall performance, recovery, or muscle anabolism [26,34,37]. It should be noted that the DS most consumed by the total sample, by sex and by performance level have solid evidence to support their efficacy [19,21,22], although this does not ensure that their use is appropriate, since each case should be studied in isolation and individually. Furthermore, although not in this order, the DS most consumed match some of those reported in other studies in rugby players [31,37] or in athletes of other sports [18,23,26,27,28].

As shown by the data found in the present study, whey protein is one of the most consumed DS in rugby players of different levels and countries [33,34,37]. In this sense, whey protein is a protein with a high biological score, which could present advantage compared to other protein sources such as casein or soy protein on an acute stimulation of muscular protein synthesis [60] and increase the rate of muscle glycogen replenishment after hard training [61]. Considering that whey protein facilitates recovery after exercise [62], its use after training and competition could optimise rugby players’ adaptation to training and sport performance [37].

Caffeine has a structure similar to adenosine and competes with it, binding to adenosine receptors A_1_ and A_2a_ [63]. Caffeine supplementation has been demonstrated to increase alertness and enhance mood and cognitive performance, reduce the rate of perceived exertion (RPE), improve cognitive performance and enhance glycolytic activity and muscle energy supply during exercise, and caffeine raises the recruitment of motor units and muscle contractility mediated by the bioavailability of intramuscular calcium [64]. In rugby, caffeine supplementation has been shown to be effective to increase performance in a battery of rugby-specific tests [65]. Moreover, the ergogenic effect of caffeine on mood and physical performance is similar in normal and elite athletes [66] and may be a suitable supplementation for amateur and professional rugby players. Nevertheless, the prevalence of caffeine supplementation in this sample is lower than that of Olympic athletes, where its consumption has been detected in 76% of them [67].

The high physical demands of rugby and equipment propitiate a dehydration that could exceed 2.0% of body mass [68], a magnitude able to impair sport performance [69]. Formulated foods and sports foods, products which provide energy and nutrients in a more convenient form than normal foods for general nutrition support or for targeted use concerning exercise (sports drinks, sports gels or sports bars) [19], are some of the DS most consumed by athletes of different ages, sex, levels or sports [18,19,24,26,27], including rugby [33,34]. Sports drinks can be a good source of simultaneous delivery of fluid with carbohydrate during exercise, as well as post-exercise rehydration and refuelling [70]. On the other hand, sports bars can be useful as a carbohydrate source during exercise and post-exercise recovery, providing carbohydrate, protein and micronutrients. Furthermore, sport drinks and sport bars could constitute an easy and suitable macronutrient intake when there is difficult access to food [19]. The intermittent dynamic of rugby requires a combination of aerobic and anaerobic energy systems, carbohydrates being an important fuel source [71]. The intake of carbohydrates amounting to 30–60 g/h enhances physical and cognitive performance in intermittent sport [72], so sport drinks and sport bars use could be good practice for rugby players.

In accordance with different studies with rugby players [33,37], creatine monohydrate is another of the DS most consumed in this study. Creatine supplementation is considered as an ergogenic aid in rugby players [73], as it promotes ATP resynthesis [74] and contributes to regulating intracellular acid–base balance regulation [75], an important function addressing the intermittent dynamics of rugby. Furthermore, creatine supplementation favours the stimulation of muscle protein synthesis and the stabilisation of biological membranes [76].

In addition to the previously mentioned DS, rugby players, due to the characteristics already commented on about their sport modality, there is scientific support that they could improve their sporting performance with other ergogenic aids [19,21,22], which have had a very low prevalence of consumption as is the case of β-alanine (4.2%), sodium bicarbonate (1.4%) and beet juice (0%). Thus, supplementation with B-alanine increases muscle carnosine synthesis, favouring the regulation of the acid–base balance at the intramuscular level and enhancing muscle contractility [77,78,79]), its ergogenic effect on efforts with a high glycolytic component having been demonstrated [80,81], while sodium bicarbonate (due to an improvement in the regulation of extracellular pH and the consequent delay in fatigue) [82,83]) has an ergogenic effect in intermittent endurance tests [84] and muscular endurance [85]. On the other hand, supplementation with beet juice is a nutritional source rich in nitrate that can increase the bioavailability of nitric oxide, improving the performance of muscle strength [86], endurance capacity [87] and intermittent high- intensity efforts [88].

Despite the high prevalence of BCAAs in this study, there is not sufficient evidence concerning the ergogenic effect of BCAA supplementation to promote muscular anabolism and performance [89,90] or increase endurance capacity [91], and it is considered to be a DS without strong evidence of its efficacy [19,21,22]. It seems that the consumption of BCAA is due to possible stimulation of protein, blood glucose insulin adjustment and synthesis and neural function improvement [62]. Moreover, it has been proposed that central fatigue is caused by a decrease in plasma BCAA that increases the tryptophan level, this being a precursor of serotonin [92]. However, more evidence is necessary. Similarly, although the prevalence of glutamine consumption is high in the present study, its consumption is currently not supported, since it is considered a DS with a deficient level of evidence [19,21,22]. Thus, glutamine supplementation has not been reported as having a positive effect on strength training [93] or on the immune function [54,94].

Though the use of supplements is widespread, it is important that both athletes and the different professionals who work with them know how to perform a cost–benefit analysis on their appropriate and responsible use [20,95], based on their individual safety, efficacy, and legality [96]. Since athletes often use DS without a clear understanding of their effects and risks, it is essential to provide information on patterns of DS use in athletes [27,97]. Furthermore, it is important for the use of DS not to compensate poor food choices and an inappropriate diet [20]. All this information could help provide nutrition education approaches that reduce the risk associated with and better use of supplementation.

The current investigation has several limitations that should be discussed to correctly apply the outcomes in competitive rugby. Although we used a validated and reliable questionnaire to assess the use of dietary supplements in elite athletes, this tool collects self-reported information in a retrospective manner, which might have induced some error due to imprecision in the number and type of supplements reported. In addition, the questionnaire was collected in the beginning of the season, which precludes the obtaining of in-season variations in the number and type of supplements used. Furthermore, although the questionnaire did not collect any information that permitted the identification of the rugby player, it is possible that some athletes may have intentionally avoided reporting some information regarding supplement consumption due to the possibility of inadvertent doping, particularly in the subgroup of professional rugby players. Finally, it is must be considered although the sample is extensive, it only corresponds to a representative sample of the Madrid community, so care must be taken to extrapolate the conclusions to all federated rugby players.

## 5. Conclusions

The prevalence of DS consumption by rugby players is 65.3%, being higher in men and in professional players. The main motivation is performance improvement, and the main places to purchase them are on the Internet and in specialised stores. Regarding the period of consumption, the majority of athletes declared consuming DS during training and in competition, the most common time being post-exercise. Concerning the most consumed DS, it was found that men and professional athletes had a higher prevalence of whey protein and creatine moohydrate consumption compared to women and amateur athletes.

## Figures and Tables

**Table 1:** Characteristics data of the participants.

Variable	Sex		Level of Performance	
	Male (*n* = 83)	Female (*n* = 61)	Professional (*n* = 69)	Amateur (*n* = 75)
Height (m)	1.80 ± 0.14	1.68 ± 0.06	1.74 ± 0.09	1.76 ± 0.15
Weight (kg)	93.0 ± 14.0	67.8 ± 10.7	24.7 ± 4.4	86.6 ± 17.6
Age (years)	24.3 ± 5.0	24.0 ± 4.9	23.5 ± 4.8	24.6 ± 5.1

Data shown as Mean (M) + Standard Deviation (SD).

**Table 2:** Dietary supplements with a higher consumption divided by sex and level of performance.

Dietary Supplement	Total (*n* = 144)	Male (*n* = 83)	Female (*n* = 61)	*p*-Value	OR	Professional (*n* = 69)	Amateur (*n* = 75)	*p*-Value	OR
Whey protein	44% (63)	58% (48)	25% (15)	< 0.001 *	1.76 [1.32–2.35]	67% (46)	23% (17)	< 0.001 *	2.57 [1.76–3.76]
Caffeine	42% (60)	43% (36)	39% (24)	0.733	1.07 [0.81–1.42]	42% (29)	41% (31)	1.000	1.02 [0.72–1.44]
Sport drinks	38% (55)	62% (34)	38% (21)	0.489	1.12 [0.85–1.49]	42% (29)	35% (26)	0.394	1.17 [0.83–1.65]
Sport bars	34% (49)	57% (28)	43% (21)	1.000	0.99 [0.73–1.33]	36% (25)	32% (24)	0.603	1.10 [0.77–1.57]
Creatine monohydrate	31% (45)	43% (36)	15% (9)	< 0.001 *	1.69 [1.30–2.18]	42% (29)	21% (16)	0.011 *	1.60 [1.15–2.21]
BCAAs	19% (27)	20% (17)	16% (10)	0.667	1.10 [0.82–1.47]	15% (21)	4% (6)	< 0.001 *	1.90 [1.41–2.56]
Glutamine	12% (17)	17% (14)	5% (3)	0.036 *	1.52 [1.15–1.99]	16% (11)	8% (6)	0.196	1.42 [0.95–2.12]

* Statistical difference in the consumption between groups (*p* < 0.05).

## Data Availability

The data presented in this study are available on request from the corresponding author. The data are not publicly available due to restrictions privacy.
